# Defensive Venoms: Is Pain Sufficient for Predator Deterrence?

**DOI:** 10.3390/toxins12040260

**Published:** 2020-04-17

**Authors:** Crystal N. Niermann, Travis G. Tate, Amber L. Suto, Rolando Barajas, Hope A. White, Olivia D. Guswiler, Stephen M. Secor, Ashlee H. Rowe, Matthew P. Rowe

**Affiliations:** 1 Department of Biology, Sam Houston State University, Huntsville, TX 77340, USA; crystal.niermann@gmail.com (C.N.N.); tray19240@gmail.com (T.G.T.); 2 Department of Integrative Biology, Michigan State University, East Lansing, MI 48824, USA; ambersuto@gmail.com (A.L.S.); oliviaguswiler@gmail.com (O.D.G.); 3 Neuroscience Program, Michigan State University, East Lansing, MI 48824, USA; rolando.barajas91@gmail.com (R.B.); hope.a.white@outlook.com (H.A.W.); 4 Department of Biological Sciences, University of Alabama, Tuscaloosa, AL 35487, USA; ssecor@ua.edu; 5 Department of Biology, University of Oklahoma, Norman, OK 73019, USA; ahrowe@ou.edu

**Keywords:** antipredator, aversive conditioning, *Centruroides*, grasshopper mouse, honest advertising, nociception, *Onychomys*, scorpion, toxicity

## Abstract

Pain, though unpleasant, is adaptive in calling an animal’s attention to potential tissue damage. A long list of animals representing diverse taxa possess venom-mediated, pain-inducing bites or stings that work by co-opting the pain-sensing pathways of potential enemies. Typically, such venoms include toxins that cause tissue damage or disrupt neuronal activity, rendering painful stings honest indicators of harm. But could pain alone be sufficient for deterring a hungry predator? Some venomologists have argued “no”; predators, in the absence of injury, would “see through” the bluff of a painful but otherwise benign sting or bite. Because most algogenic venoms are also toxic (although not vice versa), it has been difficult to disentangle the relative contributions of each component to predator deterrence. Southern grasshopper mice *(Onychomys torridus)* are voracious predators of arthropods, feeding on a diversity of scorpion species whose stings vary in painfulness, including painful Arizona bark scorpions *(Centruroides sculpturatus)* and essentially painless stripe-tailed scorpions *(Paravaejovis spinigerus)*. Moreover, southern grasshopper mice have evolved resistance to the lethal toxins in bark scorpion venom, rendering a sting from these scorpions painful but harmless. Results from a series of laboratory experiments demonstrate that painful stings matter. Grasshopper mice preferred to prey on stripe-tailed scorpions rather than bark scorpions when both species could sting; the preference disappeared when each species had their stingers blocked. A painful sting therefore appears necessary for a scorpion to deter a hungry grasshopper mouse, but it may not always be sufficient: after first attacking and consuming a painless stripe-tailed scorpion, many grasshopper mice went on to attack, kill, and eat a bark scorpion even when the scorpion was capable of stinging. Defensive venoms that result in tissue damage or neurological dysfunction may, thus, be required to condition greater aversion than venoms causing pain alone.

## 1. Introduction

The discipline of toxinology is characterized by a number of (at least partially) heuristic dichotomies. Poisons, for example, are toxins that must be ingested, inhaled, or absorbed through the skin to harm their targets, while venoms inflict damage by being injected via a bite or a sting [1,2]. Furthermore, venoms, although implicated in a range of functions from being antimicrobial [3] to aiding in intrasexual combat [4], are typically classified as serving either to increase the feeding efficiency of the venomous animal or in deterring that animal’s own enemies; i.e., in the binary roles of predation or defense [5,6,7,8,9]. These dual selection pressures have been compared broadly across taxonomic groups, with predation frequently cited as driving venom evolution and venom variability in cone snails [10,11] and snakes [12,13,14] (but see [15,16]), while predator deterrence is typically invoked to explain the venoms of echinoderms [17], bony fishes, and rays [18]. The diversity of the natural world, however, is unlikely to be fully captured by simple dichotomies, so we should not be surprised that some cone snails [10], assassin bugs [19], and no doubt many other species not only produce both predatory toxins and defensive toxins, but also deploy them selectively in the appropriate contexts of feeding vs. deterrence.

What makes for a productive predatory venom relative to an efficacious defensive venom? Coarsely and again dichotomously, the main axes deal with immobility vs. pain. Venoms used offensively, for feeding, appear designed to quickly subdue and even paralyze the prey item, minimizing its chance of escape [6,13,19]. Venoms used to deter a potential predator, in contrast, typically cause immediate, intense pain at the site of the bite or sting [1,5,9,20], leading the predator to drop or otherwise disengage from the prey, enhancing the prey’s probability of surviving the encounter [21,22,23]. Here again, absolute distinctions between venom types and functions warrant caution [24], as paralytic toxins are employed by some cone snails as a defense against their fish and cephalopod predators [10], while certain buthid scorpions deploy mammal-specific neurotoxins that disrupt muscle function in these vertebrate enemies [25,26]. These caveats aside, we are unaware of any algogenic venoms having evolved for predatory purposes, as pain is unnecessary for immobilizing one’s prey [27]. Indeed, in the context of feeding, venom components designed to cause immediate pain might be maladaptive if such toxins are costly to produce or render subjugation of the prey more difficult [23]. In sum, acute, instantaneous pain appears intrinsic to defensive bites and stings [1,5,23].

Immediate pain, however, may not be the only ingredient needed for an effective defensive venom. So too is damage, what Nelsen et al. [2] (p. 451) might refer to as “pathophysiological injury.” Unlike pain, such injury need not be immediate but can unfold in the minutes, hours, days, or longer following a sting or bite from a venomous animal; in humans, dramatic examples include tissue necrosis associated with many viper bites [28] and the cardiac and respiratory failure resulting from the stings of certain buthid scorpions [29]. Death, of course, is the ultimate injury, although in most cases involving prey interacting with their natural predators the damage is nonlethal [5], in part due to coevolution of venom toxicity and venom resistance between the hunters and the hunted [15,30,31,32,33]. The important point, argued by some venomologists, is that defensive bites and stings must cause some harm lest a predator learn the painful bite or sting is simply a bluff and presses home its attack [22,34,35,36]. According to Schmidt, “pain is the advertisement, and toxicity is the truth” [35] (p. 45). This perspective that pain, as a signal, must honestly reflect the dangerousness of a venomous organism is functionally similar to the better-studied, though still unresolved, argument of whether the warning colors of poisonous organisms such as poison dart frogs, heliconid butterflies, and ladybird beetles are honest indicators of these animals’ toxicity [37].

Surprisingly, given their presumed importance, defensive venoms have been poorly studied [20,22,38]. Only a handful of investigations have demonstrated the efficacy of such venoms against natural predators [21,38,39,40,41,42,43]. Moreover, no study has attempted to tease apart the relative contributions of the painful vs. toxic elements to a venom’s effectiveness in deterring an enemy, perhaps because most defensive cocktails contain both components [23]. Fortuitously, an arms race between a carnivorous mouse and its scorpion prey provides the opportunity to test the hypothesis that pain alone, in the absence of venom toxicity, is sufficient to turn away a potential predator.

The interaction involves Arizona (AZ) bark scorpions (*Centruroides sculpturatus*, formerly known as *C. exilicauda*) and southern grasshopper mice (*Onychomys torridus*) in the Sonoran Desert. Among North American scorpion species, AZ bark scorpions are known for inflicting painful as well as potentially lethal stings [44,45,46,47]. Bark scorpion venom is a cocktail of low-molecular-weight proteins that bind sodium (Na^+^) and potassium (K^+^) ion channels in sensory neurons, motor neurons, and muscle tissue [48,49,50,51]. Anecdotal reports describe bark scorpion stings as producing immediate burning pain, followed by intense, long-lasting, throbbing pain coupled with hypersensitivity to touch and pressure. Southern grasshopper mice are diminutive but voracious carnivores that readily consume AZ bark scorpions [21]. While grasshopper mice are stung during attacks on bark scorpions, the mice have evolved resistance to both the lethal and pain-inducing toxins in the scorpion’s venom [21,52].

The mechanism of the mice’s resistance to bark scorpion venom involves mutations in their voltage-gated Na^+^ channels (Na_v_), the targets of the scorpion toxins. Mammals possess nine isoforms of these channels, Na_v_1.1 to Na_v_1.9, expressed differentially across various tissues [53,54]. Na_v_1.4 is expressed in skeletal muscle and is critical to normal muscle function. Envenomation from scorpions in the genus *Centruroides* are potentially lethal because their toxins disrupt enervation to the mammalian diaphragm, leading to asphyxiation [55]. Southern grasshopper mice possess several mutations in their Na_v_1.4 that effectively neutralize the lethal toxins in bark scorpion venom [56], providing a level of resistance nearly 30 times greater than that of a lab mouse *(Mus musculus)* [57]. Arizona bark scorpions are the only scorpion of medical significance in the United States, with children under two years of age at risk of death from a single encounter [46,47,58]. In contrast, pilot studies using staged encounters have shown that a southern grasshopper mouse will feed to satiation on *C. sculpturatus*, voluntarily attacking, getting stung by, and consuming up to six AZ bark scorpions in a row, representing >10% of the body mass of the mouse, without such risk [59]. In short, southern grasshopper mice are exceptionally resistant to the toxic components of AZ bark scorpion venom.

Southern grasshopper mice are also resistant to the algogenic components in AZ bark scorpion venom, again because of mutations to one of their ion channels. While several such channels are involved in regulating pain signals, Na_v_1.7 and Na_v_1.8 are critical for initiating and propagating nociceptive action potentials, or APs [60,61]. Noxious stimuli induce generator potentials that activate Na_v_1.7 and depolarize nociceptors, firing Na_v_1.8. Activation of Na_v_1.8 generates the Na^+^ current needed to propagate signals to the central nervous system (CNS) [60,61]. In sensitive animals, bark scorpion venom binds Na_v_1.7, recruiting Na_v_1.8 and initiating prolonged APs [62]. In grasshopper mice, as well, bark scorpion venom initiates AP firing via activation of Na_v_1.7. However, amino acid variants in grasshopper mice Na_v_1.8 bind venom proteins, inhibiting Na^+^ currents and blocking APs. Thus, grasshopper mice have evolved structural modifications to Na_v_1.8 that co-opt venom proteins to block transmission of venom-initiated pain signals to the CNS; AZ bark scorpion venom, in essence, acts as an analgesic when southern grasshopper mice get stung [52].

Grasshopper mice, however, are not completely insensitive to the painful stings of bark scorpions. Injections of bark scorpion venom into a grasshopper mouse’s hind paw (a standard measure of sensitivity to an aversive stimulus) elicit approximately eight seconds of paw licking before the painkilling effects of the venom are realized [52]. This short window of pain, coupled with the mice’s resistance to the debilitating toxins of AZ bark scorpion venom, provides a conservative test of whether pain, by itself, influences the predatory behavior and prey preferences of grasshopper mice when feeding on scorpions.

*Centruroides scuplturatus* is not the only species of scorpion available to grasshopper mice when they go hunting. The stripe-tailed scorpion (*Paravaejovis spinigerus*, formerly known as *Vaejovis spinigerus*) is another relatively abundant species of scorpion sympatric with both southern grasshopper mice and AZ bark scorpions in the Sonoran Desert [63]. Our previous work showed that stripe-tailed scorpion venom is slightly irritating but significantly less painful than bark scorpion venom to house mice *(Mus musculus)* [62]. We also demonstrated that grasshopper mice, in staged encounters, expend more time and effort to subdue bark scorpions compared with stripe-tailed scorpions [21]. The mice never respond to stings from stripe-tailed scorpions, but often respond to stings from bark scorpions by flinching and sometimes grooming the site of the sting [21]. Additionally, grasshopper mice drop bark scorpions more often than stripe-tailed scorpions, necessitating multiple attacks and longer latencies to subdue bark scorpions [21]. Indeed, grasshopper mice show no more difficulty in attacking and subduing a stripe-tailed scorpion than they do when feeding on defenseless house crickets *(Acheta domesticus)* [21]. Thus, when grasshopper mice are foraging, they likely have a choice between scorpion species that differ in pain-inducing capability; and pain, albeit brief, may influence that selection.

We tested this hypothesis by training wild-caught and captive-bred grasshopper mice to feed on a scorpion restrained in a small cup. Following training, we offered each mouse a simultaneous choice between two cups: one containing a bark scorpion whose sting is briefly painful to the mice; the second holding a stripe-tailed scorpion whose sting is painless to the rodents. Our protocol permitted us to examine the preference of grasshopper mice at each stage of a predatory sequence, including search, pursuit, attack, and consumption [64,65] (Table 1). Our results show that grasshopper mice distinguish between scorpion species, and that the mice prefer species with less painful stings. When both species are rendered painless, however, grasshopper mice seemingly base their choice on the scorpion’s nutritional value.

## 2. Results

### 2.1. Mice Prefer to Pursue, Attack, and Consume Less-Painful Scorpions

Assessment of the factors influencing a grasshopper mouse’s preference for AZ bark scorpions or stripe-tailed scorpions required three separate experiments. Our intent in Experiment 1 was to mimic a natural encounter between scorpions and mice. Because adult stripe-tailed scorpions are larger than adult bark scorpions (Table S1), scorpions were selected randomly from their containers, generating a size difference between the bark scorpions and the stripe-tailed scorpions. Experiment 2 controlled for the body mass of scorpions from both species but allowed each species to sting normally. Experiment 3 controlled for both body mass and sting painfulness by gluing a small tube over the stinger of each species. See Table 2 for more details regarding the objectives of each experiment, the manipulations involved, and the statistical results comparing the body masses of the individual scorpions from both species used in a given experiment.

#### 2.1.1. Experiment 1

**Figure 1:**
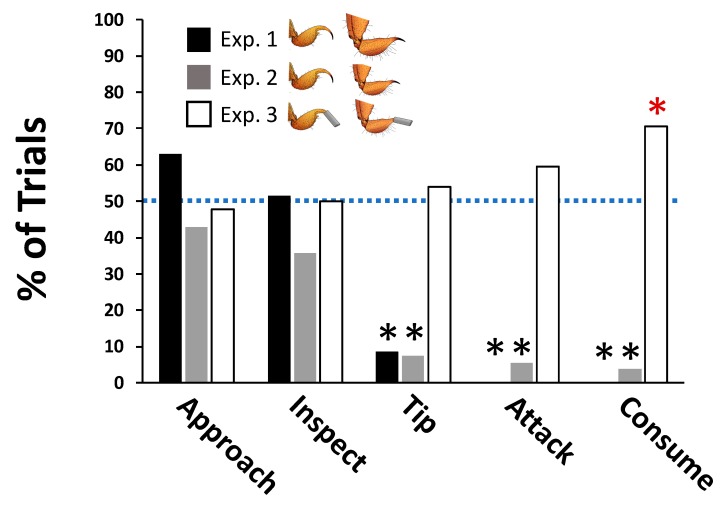
The mean percent of trials southern grasshopper mice selected the AZ bark scorpion before the stripe-tailed scorpion for each of the five dependent variables (DVs) in three experiments. Experiment 1 used adult scorpions of average size (stripe-tailed scorpions are larger than AZ bark scorpions) capable of stinging. Experiment 2 used equally sized scorpions of both species, again with functional stingers. Experiment 3 used equally sized scorpions of both species that had their stingers covered by a tiny tube and, thus, could not sting. Bars overscored with a black * demonstrate a significant preference (*p* < 0.00001) by the mice for first tipping, attacking, and consuming a striped-tailed scorpion when both species of scorpions could sting. The single bar overscored by a red * reveals a significant preference (*p* = 0.004) by the mice for consuming AZ bark scorpions when neither species could sting. The dashed blue line represents the expected value if grasshopper mice were randomly selecting a species of scorpion.

In Experiment 1, when the grasshopper mice were simultaneously presented with two species of scorpion, one larger and less painful than the other, they exhibited a clear preference beginning with the pursuit stage (i.e., tip; Table 1) of the predatory sequence. Figure 1 shows the frequency with which grasshopper mice, collectively and on average, selected the bark scorpion over the stripe-tailed scorpion for each of our five dependent variables. Mice first approached and inspected each species with nearly equal, statistically indistinguishable frequency (Figure 1). Following this examination, however, the mice made an unambiguous choice. Mice first tipped the cup containing a smaller, more painful bark scorpion only 8.6% of the time, demonstrating a highly significant, collective preference for stripe-tailed scorpions (Z_s_ = 3.896, *p* < 0.0001; Figure 1). The mice were equally selective after tipping the cup, preferring to first attack and consume the larger and less-painful stripe-tailed scorpion (Z_s_ = 4.896, *p* < 0.0001; Figure 1). See Table S5 for the choices made by individual mice across each of their six trials for all five dependent variables (DVs) in Experiment 1.

#### 2.1.2. Experiment 2

Experiment 2 employed a different group of laboratory born mice but controlled for the size differences between the two scorpion species while permitting each to sting normally. Here too the mice collectively and consistently, in the latter stages of the predatory sequence, preferred the painless stripe-tailed scorpion over the painful bark scorpion even though both were of equal mass. Grasshopper mice first tipped the cup containing a bark scorpion in only 7.41% of the trials (Z_s_ = 4.979, *p* < 0.0001; Figure 1), attacked the bark scorpion first in just 5.56% of the interactions (Z_s_ = 5.229, *p* < 0.0001; Figure 1), and first started eating the bark scorpion in a mere 3.7% of the encounters (Z_s_ = 5.479, *p* < 0.0001; Figure 1). See Table S6 for the choices made by individual mice across each of their eight trials for all five DVs in Experiment 2.

#### 2.1.3. Experiment 3

Choice results from Experiment 3, using a different set of laboratory-born mice, contrast with those from the previous two experiments. When selecting between two equally sized species of scorpions rendered stingless, the mice showed no collective preference for first approaching, inspecting, tipping, or attacking either species (Figure 1). They did, however, exhibit a significant collective preference for first consuming the bark scorpion (Z_s_ = 2.854, *p* = 0.004, Figure 1). See Table S7 for the choices made by individual mice across each of their 10 trials for all five DVs in Experiment 3.

### 2.2. Mice Are More Cautious When Pursuing Scorpions with Painful Stings

The caution with which grasshopper mice approach a predatory interaction is also revealed in whether they inspected just one of the two cups before tipping it or chose to inspect both cups before selecting one to tip. In Experiments 1 and 2, when scorpions could sting, the mice rarely made the decision to inspect just one cup before their first tip (7/35 trials = 20% and 7/56 trials = 12.5%, respectively). In Experiment 3, with both species of scorpions rendered harmless, the mice appeared less cautious, with “one-cup inspections” accounting for 33.3% (30/90) of the trials. A test of proportions comparing results from Experiments 2 and 3 is particularly informative. While Experiment 1 used a mix of wild-caught and captive-born grasshopper mice, the subjects in Experiments 2 and 3 were all laboratory born and, thus, equally inexperienced with scorpions. The proportion of trials where the mice chose to tip a cup before inspecting both cups was significantly higher when they were preying on defenseless scorpions (test of proportions, z = 2.814, *p* = 0.005).

### 2.3. Painful Stings Inhibit but Do Not Prevent Mice from Making Subsequent Attacks

Inferences regarding the feeding preferences of grasshopper mice are revealed not only by their first choices when simultaneously presented with both species of scorpions, but also in their subsequent willingness to prey on the second scorpion in the arena. In Experiment 3, when given the choice between equal-sized, “stingless” scorpions of both species, the mice frequently turned their attention to the remaining scorpion. The mice were more reluctant, however, in Experiments 1 and 2 when they were dealing with scorpions capable of stinging. Here again, a comparison of results from Experiments 2 and 3 is instructive. Grasshopper mice were significantly more likely to tip a second cup (test of proportions, z = 4.649, *p* < 0.0001) when neither species of scorpion could sting (77/90 trials = 85.6%, Experiment 3) than when the second cup contained a bark scorpion capable of inflicting its briefly painful sting (28/56 trials = 50%, Experiment 2). Similarly, the mice were significantly more likely to attack a second scorpion (test of proportions, z = 6.01, *p* < 0.0001) when both had been rendered harmless (77/90 trials = 85.6%, Experiment 3) than when the second choice was a fully armed bark scorpion (21/56 trials = 37.5%, Experiment 2).

Interestingly, there were also differences between mice in Experiments 1 and 2 even though both groups were dealing with scorpions capable of stinging. The mix of wild and short-term captive mice in Experiment 1 were significantly more likely than longer-term captive mice used in Experiment 2 to both tip (27/35 trials = 77.1% vs. 28/56 trials = 50%, respectively; z = 2.56, *p* = 0.01) and attack (24/35 trials = 68.6% vs. 21/56 trials = 37.5%, respectively; z = 2.884, *p* = 0.004) the remaining bark scorpion.

### 2.4. Grasshopper Mice Appear Not to Anticipate the Location of Painful Prey

An unexpected result from all three experiments was the lack of choice exhibited by the mice during the search stage (Table 1) of a predatory encounter. Collectively, the mice showed no preference for which species of scorpion they first approached or first inspected (Figure 1). Individually, only two individuals out of the 22 mice in the study demonstrated a significant preference during these early stages: one mouse in Experiment 2 first inspected the stripe-tailed scorpion (Table S6), while another mouse in Experiment 3 first approached the AZ bark scorpion (Table S7), results conceivably explained by chance. Given that the position in the arena of the AZ bark scorpion and stripe-tailed scorpion remained constant for any given mouse during both its training trials and its preference tests, we presumed the mice might learn which corner was occupied by a painful AZ bark scorpion and which corner harbored a harmless stripe-tailed scorpion, directly approaching and inspecting the latter while avoiding the former. The absence of a significant preference by the mice during the early search stages of a predatory interaction might have two explanations. Prey items in resource-poor environments like the Sonoran Desert may be encountered so irregularly that it would be difficult for grasshopper mice to predict where and when a prey item might appear. Alternatively, the possibility of encountering an AZ bark scorpion with a painful sting might cause a grasshopper mouse to base its choice of which scorpion to first attack on confirmatory evidence (possibly odor cues [66]) provided only by a close inspection of both cups in adjacent corners of the arena, overriding decisions that might have been made earlier in the predatory sequence.

### 2.5. Handling Times Increase with Sting Painfulness

**Figure 2:**
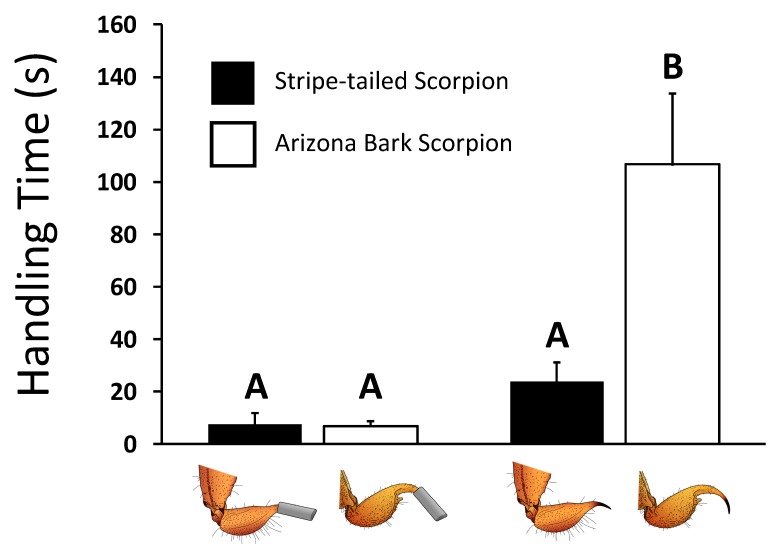
Mean (+ 1 SE) elapsed duration between the first attack of a grasshopper mouse and the eventual incapacitation of the scorpion (i.e., handling time) as a function of whether the scorpion could or could not sting. Histograms overscored by the same letter failed to differ at the *p* < 0.05 level of significance.

Results from the mixed-model ANOVA demonstrated that grasshopper mice took longer to subdue bark scorpions than stripe-tailed scorpions (F_1, 16.826_, F = 27.789, *p* < 0.0001), and longer to bring down scorpions capable of stinging (F_1, 18.517_, F = 37.060, *p* = < 0.0001). Both main effects, however, were driven by their significant interaction (F_1, 16.826_, F = 29.453, *p* < 0.0001). Confidence intervals revealed handling times were significantly longer (*p* < 0.05) for grasshopper mice interacting with bark scorpions capable of stinging (handling time (mean ± SE) = 106.5 ± 27.1 s) than when dealing with similarly capable striped-tailed scorpions (23.7 ± 7.3 s), stingless bark scorpions (6.9 ± 1.7 s), or stingless stripe-tailed scorpions (7.5 ± 4.3 s), which themselves did not differ (Figure 2). The effect size for the model was r^2^ = 0.689. Data used for the analysis of handling times can be found in Table S8.

### 2.6. Arizona Bark Scorions Have a Higher Energy Content

The per-gram energy content of AZ bark scorpions is significantly greater than for stripe-tailed scorpions, in both wet weight kJ/g (Arizona bark scorpions = 10.1 ± 0.37; stripe-tailed scorpions = 7.6 ± 0.23; t = 6.07, DF = 62, *p* < 0.0001; r^2^ = 0.373) and dry weight kJ/g (Arizona bark scorpions = 25.0 ± 0.17; stripe-tailed scorpions = 23.5 ± 0.24; t = 4.43, DF = 62, *p* < 0.0001; r^2^ = 0.240). The calorimetry data for these analyses are available in Table S1.

## 3. Discussion

Our goal was to test the hypothesis that painful but nontoxic venoms can deter a potential predator. Our use of southern grasshopper mice feeding on AZ bark scorpions provided a conservative test of the hypothesis, as the mice have evolved resistance to the algogenic components in the scorpion’s venom; a sting that can cause intense pain that lasts for hours in humans generates only a few seconds of mild irritation for a grasshopper mouse [52]. Our results nonetheless demonstrate that even brief pain matters to grasshopper mice, at least when they have access to alternative prey.

Experiment 1 was designed to mimic, to the best of our ability, the situation a grasshopper mouse might experience in the field when encountering a larger, minimally or nonpainful, adult stripe-tailed scorpion or a smaller, significantly more painful, adult bark scorpion. Both collectively (Figure 1) and individually (Table S5), the mice first chose to pursue (tip), attack, and consume the stripe-tailed scorpion. Either the larger body mass of the stripe-tailed scorpions or their less painful stings, or both, may have contributed to the mice’s preference. Results from the remaining two experiments, each controlling for scorpion body size, point more directly to the bark scorpion’s briefly painful sting as the deciding factor.

Indeed, results from Experiment 3 suggest that bark scorpions might be nutritionally more rewarding for a grasshopper mouse once the painfulness of its sting, and the greater handling costs such a sting imposes, have been removed from the equation. When presented with stingless scorpions matched in size, grasshopper mice collectively (Figure 1) preferred to first eat the bark scorpion (and frequently did so individually; see Table S7), even though there was no preference for either scorpion species in the search, pursuit, or attack stages of the predatory sequence. Such energetic benefits are devalued, however, when bark scorpions can deliver their irritating sting. Results from Experiment 2, employing equal-sized scorpions of both species, each capable of stinging, demonstrate that grasshopper mice, both individually (Table S6) and collectively (Figure 1), prefer to pursue, attack, and consume the less energetically rewarding but less painful stripe-tailed scorpion. Southern grasshopper mice, even with their remarkable physiological resistance to the algogenic toxins in bark scorpion venom, choose to avoid the pain when alternate prey are available.

The importance of a painful sting to grasshopper mice was evinced not only by their propensity to first attack the painless stripe-tailed scorpion when both species of scorpions could sting, but also by their greater caution when making that decision; e.g., when faced with two species of same-sized, fully armed scorpions, grasshopper mice were significantly more likely to inspect both cups rather than just one prior to pressing an attack, appearing to “double-check” their choice lest they tip a cup containing an AZ bark scorpion with a painful sting. The consequence of pain was also revealed in the willingness of the mice to pursue the remaining scorpion, typically a bark scorpion, after consuming the first: mice were significantly more likely to attack the second scorpion when both species had been rendered stingless.

Pain therefore appears necessary to deter a hungry grasshopper mouse—but it was not always sufficient. Although the mice were less likely to return for a second meal when it was an AZ bark scorpion capable of stinging, the deterrence of a painful sting was not absolute. Our first experiment employing a mix of wild-caught and captive-bred mice showed they had a significant preference for painless stripe-tailed scorpions over the momentarily painful AZ bark scorpions. But the mice, after first eating the stripe-tailed scorpion, went on to tip the cup containing the bark scorpion roughly three times out of four, attacking the bark scorpion in over two-thirds of the trials, and eating the bark scorpion nearly half the time. Experiment 2 used only captive-born grasshopper mice, yet the results were similar; i.e., after feeding on the harmless stripe-tailed scorpion, the mice often turned their attention to the troublesome bark scorpion, tipping its cup half the time while attacking and consuming the bark scorpion in one-third of the trials. The greater reluctance to attack a bark scorpion shown by grasshopper mice in the second experiment may reflect “state-dependent foraging” [67,68] as the average mass of these seven captive-born and long-term captive mice (28.9 ± 1.09 g) was over 20% heavier than the mass of the four wild-caught and two short-term, captive-born mice (23.6 ± 1.55 g) used in the first experiment, a significant difference (t = 2.856, DF = 11, *p* = 0.016) (see Table S9 for the masses of individual mice used in each of the three experiments). After seven months of ad lib mouse chow, well-fed grasshopper mice appear to lose some but not all interest in feeding on scorpions possessing painful stings.

The temporary protection provided by algogenic toxins may explain why defensive venoms are typically both painful and toxic. For a venomous prey animal, an important benefit of delivering a painful bite or sting can be the immediate disengagement of its predator (e.g., [21]). That pain can also generate more enduring avoidance by an enemy is evident from the long history of operant learning experiments employing electric shocks to train laboratory rats and mice (beginning with [69], made famous by Skinner [70], and still in use today [71]). A consistent result from these studies is that greater aversion is conditioned by higher intensities of shock [72,73]. By extension, possession of venom constituents inflicting tissue damage or neuronal dysfunction might condition even stronger aversion than peripheral pain alone, increasing the latency before a predator again attacks that venomous prey [74]. The degree to which pain and toxicity act synergistically to influence aversive conditioning in predators awaits more detailed investigation, but two disparate lines of evidence provide support for such an interaction. Sheep find familiar foods mildly aversive when infused with a novel odor but react with significantly greater aversion when the experimental food also induces toxicosis [75,76]. Neurologically, the various nociceptive signals that alert animals to danger (including distastefulness, toxicosis, and peripheral pain, as potentially associated with a bite or sting) are processed in the same region of the brain, the parabrachial nucleus (PbN), which plays an important role in aversive conditioning [77,78]. Thus, while immediate pain appears essential for interrupting a given attack by a predator, both pain and toxicity may contribute to training the predator to avoid the prey item in the future.

The list of organisms employing venom-mediated, pain-inducing bites and stings is large and diverse. Co-opting the pain-signaling pathways of potential enemies appears to be a successful evolutionary strategy, even though few studies have demonstrated the fitness benefits of possessing a painful sting or bite. Our results add to the short list of such studies, demonstrating that the more painful stings delivered by AZ bark scorpions shift the predatory behavior of southern grasshopper mice towards less painful prey, in this case to stripe-tailed scorpions. The benefits to the bark scorpion are dynamic, however, and not unconditional. While southern grasshopper mice might, in the wild, forgo feeding on AZ bark scorpions when prey are plentiful and the mice well fed, bark scorpions would be back on the menu when prey are scarce and the mice are malnourished. We suggest a potentially productive avenue of research will be to examine the proximate causes of such state-dependent aversion. What neurological, physiological, and genetic mechanisms trigger a well-fed grasshopper mouse to avoid interacting with a painful AZ bark scorpion, while a nutritionally stressed mouse will ignore the pain and attack? Intriguingly but perhaps not surprisingly, the PbN appears to mediate the conflicting demands of pain and hunger in lab mice *(Mus musculus)*; neurotransmitters released in the PbN of a food-deprived mouse reduce its sensitivity to inflammatory pain in its paws, potentially permitting the mouse to continue foraging [79]. Fruit flies *(Drosophila melanogaster)* face a similar dilemma when deciding whether or not to feed on poisonous, bitter-tasting foods; in this case, specialized taste neurons that normally inhibit ingestion of bitter tastants in well-fed flies are turned off in hungry flies, enabling them to avoid starvation by feeding on more dangerous foods [80]. The inclusion of AZ bark scorpions in the diet of hungry grasshopper mice may be mediated by similar mechanisms in their PbN or, perhaps, by downregulation of Na_v_1.7 and upregulation of Na_v_1.8 in their nociceptors, rendering a briefly painful sting even less consequential.

## 4. Conclusions

Because venoms used defensively are typically both painful and toxic, it has been difficult to isolate their relative contributions to predator deterrence. The fortuitous effects of an arms race between carnivorous, southern grasshopper mice and their AZ bark scorpion prey provide a conservative test of the importance of pain. The mice are completely resistant to the toxic components in bark scorpion venom, and partially resistant to the toxins that produce pain; a sting that can cause hours of intense discomfort in humans induces a brief few seconds of irritation in the mice. Nevertheless, even brief pain matters. When provided a choice between feeding on a briefly painful AZ bark scorpion or a painless stripe-tailed scorpion, both matched in body mass, the mice preferred to attack the stripe-tailed scorpion. Such deterrence, however, was short-lived, as the mice frequently then attacked, got stung by, and eventually killed and consumed the bark scorpion. Thus, the toxic components of defensive venoms, i.e., constituents causing tissue damage and/or neuronal disruption, might be necessary to condition greater aversion in an enemy.

## 5. Materials and Methods

### 5.1. Animal Care and Use Protocols

Protocols for this study were approved by the Institutional Animal Care and Use Committees at the University of Texas at Austin (UT Austin, protocol AUP-2011-00103, approved on 7 November 2011), Michigan State University (MSU, protocols 10/13-229-00 and 11/16-191-00, approved on 11 July 2013 and 11 February 2016, respectively), and the University of Oklahoma (OU, protocol R18-016, approved on 17 July 2018). The wild grasshopper mice used in Experiment 1, and that were the parents of the captive-born mice used in Experiments 1–3, were collected with the approval of the Arizona Department of Game and Fish (scientific collecting permits SP558334, SP760407, and SP591398).

### 5.2. Study Subjects

#### 5.2.1. Mice

Southern grasshopper mice were collected from the Santa Rita Experimental Range (SRER) located in the Santa Rita Mountains of southern Arizona. Sherman small-mammal live traps were baited with dry cat food, set at dusk, and checked before dawn. Mice were transported to the animal facilities at UT Austin or MSU, where they were housed in extra-large mouse cages containing bedding, material for nest construction, tubes, and running wheels. Mice were maintained on a 14/10 light/dark cycle and provided with water and mouse chow ad libitum. Their diet was supplemented with live crickets *(Acheta domesticus)* and superworms *(Zophobas morio)* for the first two months of captivity to ensure they did not lose weight while transitioning to mouse chow. Food was removed from cages the night before training trials and preference tests. Our experimental protocol involved three experiments, employing three different groups of mice. Experiment 1 used a group of six adult mice (three females, three males; mass (mean ± SE) = 23.6 ± 1.55 g). Four of the mice were wild-caught (two adults, one subadult, and one juvenile) that may or may not have experienced scorpions in the wild. The other two mice were born in captivity, had not had previous experience with scorpions, and were approximately four months old at the time of the experiments. Experiment 2 used a different group of seven mice (five females and two males) that were also born in captivity to wild-caught mice; this group of mice, scorpion-naïve until their training trials, had been maintained in captivity for approximately seven months (mass = 28.9 ± 1.09 g). Experiment 3 used a group of nine mice that were born in captivity from wild-caught parents; these mice (four females, five males) had been maintained in captivity for approximately one year before being tested (mass = 35.8 ± 1.45 g), and were also scorpion-naïve prior to training.

#### 5.2.2. Scorpions

AZ bark scorpions and stripe-tailed scorpions were collected from the SRER at night using ultraviolet light. Specimens were housed in groups, separated by species, in plastic containers (52 cm L × 35 cm W × 15.6 cm H) containing substrate (aquarium gravel) and refuge (egg crate). Scorpions were fed live crickets twice per month and provided with water ad libitum.

### 5.3. Single-Cup Training Trials

Mice were habituated to a test arena (a glass terrarium 61 cm L × 30.5 cm W × 43 cm H) and trained to tip a small, translucent plastic cup (42 mm diameter bottom × 28 mm H × 60 mm diameter mouth) to obtain a nonvenomous prey item (superworm). Grasshopper mice voraciously attack any small, moving object [66,81,82], and pilot trials using unrestrained prey demonstrated the mice would attack whatever prey happened to move first. Use of the cups limited each scorpion’s locomotion and blurred its image to the mouse, forcing mice to select prey on qualities other than just movement.

Following their experience with superworms, mice were trained to expect scorpion prey in the plastic cups. Mice were presented alternately (and in counter-balanced order) with a single cup containing either an AZ bark scorpion or a stripe-tailed scorpion. Scorpion species were placed in adjacent corners of the test chamber. For individual mice, the location of each species remained the same across trials. After placing a cup containing a scorpion into the chamber, a mouse was introduced and given 10 min to engage with the scorpion. Each mouse was provided six opportunities to interact with each species of scorpion (total trials per mouse = 12) in a systematically counterbalanced order, with three presentations per day scheduled over a period of one to two weeks. The mass of prey presented to a grasshopper mouse on any given day (in the training trials described here, or in the preference tests outlined below) was always below the average mass of live prey readily eaten by captive *O. torridus* maintained on an ad libitum diet of mouse chow [81].

### 5.4. Double-Cup Preference Tests

Following the single-cup training trials, mice were presented simultaneously with two cups, one containing an AZ bark scorpion and one containing a stripe-tailed scorpion. Scorpion species were placed in adjacent corners of the test chamber: for individual mice, the location of each species of scorpion was consistent with their training trials and remained consistent across preference tests. After placing cups containing scorpions into the chamber, a mouse was introduced and given 10 min to interact with the scorpion prey. In Experiment 1, each mouse was tested twice during each of three sessions (six trials total), two trials per day scheduled over a period of a week. To increase the statistical power of the tests, we increased the number of trials to eight total trials per mouse in Experiment 2 (two each on four different days), spread over the course of 2–3 weeks. In Experiment 3, due to a greater supply of scorpions, the number of tests on each mouse was increased to 10 (two each on five different days), distributed again over a 2–3 week period.

Adult stripe-tailed scorpions are larger than adult bark scorpions (Table S1). Our intent in Experiment 1 was to mimic a natural encounter between scorpions and mice, so scorpions were selected randomly from their containers, generating a size difference between the bark scorpions and the stripe-tailed scorpions. Experiment 2 controlled for the body mass of scorpions from both species but allowed each species to sting normally. Experiment 3 controlled for both body mass and sting painfulness by gluing a small tube over the stinger of each species. See Table 2 for more details regarding the objectives of each experiment, the manipulations involved, and the statistical results comparing the body masses of the scorpions from both species used in a given experiment.

### 5.5. Behavrioral Analyses

Both training trials and preference tests were videotaped for analysis using a Sony HDR-CX700V HD camcorder. A curtain was used to separate the camcorder and personnel from the test chamber so that mice would not be distracted during trials. We used videos from the training trials to measure the difficulty mice experienced when attempting to subjugate (i.e., handling time, Table 2) a single individual of each of the two species of scorpions when capable and incapable of stinging. Handling times were measured using BORIS video-analysis software [82]. The proficiency with which grasshopper mice subdue dangerous prey improves with experience [21,81,83]. We therefore used the first three exposures (out of six) of each mouse to each species of scorpion in the training trials to equilibrate experiences of our mix of field-caught and captive-born mice, employing the last three exposures for calculating handling times. Prey choice was assessed from videos of the preference tests using five dependent variables (DVs), each based on which species of scorpion a mouse first: approached, inspected, tipped, attacked, and consumed (Table 1).

### 5.6. Scorpion Energy Content

We determined the energy content of individual scorpions by bomb calorimetry [84]. Each scorpion was weighed fresh (wet mass), dried to a constant mass at 60 °C, and reweighed (dry mass). Dried scorpions were placed into individual gelatin capsules (size 00, 0.1152 ± 0.0002 g, *n* = 77, Electron Microscopy Science, Hatfield, PA). Each scorpion and capsule were ignited in a bomb calorimeter (model 1266; Parr Instruments Moline, IL, USA) to determine total energy content (kJ g^−1^). We subtracted from total energy the energy of each gelatin capsule (19.74 ± 0.10 kJ g^−1^, *n* = 3) to determine the energy content of each scorpion (dry mass, kJ g^−1^). Wet mass energy content (kJ g^−1^) was determined as the product of dry mass energy content and the scorpion’s dry mass percentage.

### 5.7. Statistical Analyses

Handling times were analyzed with SPSS Statistics (v. 24.0, IBM, Armonk, NY, USA) using a mixed-model ANOVA that included two within-subjects factors (trial number and scorpion species) and a single between-subjects factor (experiment number). To meet the parametric assumptions of normality and variance homogeneity, handling times were transformed using the equation log (X + 1) [85], which nonetheless failed to correct for normality. We therefore reduced our α to 0.01 [86]. As additional protection against Type 1 error, we employed the “unstructured” covariance procedure which calculates independent variances for each treatment. Results from the full model revealed insignificant differences in handling times between Experiments 1 and 2, as well as an insignificant trial effect. We therefore, in a reduced model, dropped the trial effect and compared the pooled handling times of Experiments 1 and 2 with those of Experiment 3 (generating a “sting vs. stingless” comparison). Results from the full and reduced ANOVAs were essentially identical; we here report the latter both for ease of interpretation and because the reduced model better fit the data (Akaike’s Information Criterion of 133.4 vs. 120.5, respectively). Effect size for the reduced model is reported as r^2^.

Differences in the wet and dry mass energy content across both species of scorpions were analyzed with a t-test using JMP (v. 11.0.0, SAS Institute, Cary, NC, USA). Alpha was set at 0.05, and effect size is reported as r^2^.

The dichotomous nature of our “choice” data dictated the use of nonparametric statistics for the majority of the training trial and preference test analyses. The choices made by individual mice, on each of the five DVs for each of the three preference-test experiments, were assessed using a binomial test and are reported in Tables S5–S7. These individual choices were then pooled, providing a collective measure of the mice’s preference, and tested for significance using the Z-transform test [87]. The frequency with which mice made different choices on the same DVs across different experiments was assessed using a test of proportions. Analyses were performed using JMP 11 (SAS), Social Science Stats (http://www.socscistatistics.com), and Vassarstats (http://vassarstats.net).

## Figures and Tables

**Table 1:** Operational definitions of the dependent variables corresponding to each stage of a predatory sequence measured during interactions between grasshopper mice and both species of scorpions.

Type of Trial	Stage of Predatory Sequence ^1^	Dependent Variable ^2^	Definition
Single-cup training trials		Handling Time	Elapsed time between a mouse’s first attack (see below) and the moment the scorpion was no longer capable of fighting back or escaping
Double-cup preference trials	Search	Approach	Mouse moves to within ½ body length of cup containing scorpion
	Search	Inspect	Mouse sticks nose over the top of the cup, breaking an imaginary vertical plane at the edge of the cup
	Pursuit	Tip	Mouse tips the cup on its side
	Attack	Attack	Mouse attempts to capture scorpion by lunging with forepaws outstretched and mouth open
	Consume	Consume	Mouse begins eating the scorpion

^1^ Adapted from [64,65]. ^2^ Measured from digital recordings of each trial.

**Table 2:** Objectives, scorpion masses, statistical comparisons, and stinger manipulations associated with each of three experiments designed to assess the preferences of southern grasshopper mice *(Onychomys torridus)* for feeding on painful AZ bark scorpions *(Centruroides sculpturatus)* vs. painless stripe-tailed scorpions *(Paravaejovis spinigerus)* when one individual of each scorpion species was presented simultaneously to an individual grasshopper mouse. Scorpion masses reported as mean ± SE (sample size). Effect size for the single significant parametric test is reported as r^2^. See Tables S2–S4 for body masses of the scorpions used in each of the three experiments.

	Experiment #1	Experiment #2	Experiment #3
Objective	mimic natural encounters in the field	control for scorpion mass but not sting painfulness	control for both scorpion mass and sting painfulness
AZ bark-scorpion mass	0.555 ± 0.018 g (*n* = 72)	0.784 ± 0.017 g (*n* = 98)	0.48 ± 0.010 g (*n* = 145)
Stripe-tailed scorpion mass	0.838 ± 0.031 g (*n* = 72)	0.784 ± 0.017 g (*n* = 98)	0.47 ± 0.010 g (*n* = 143)
Mass differences between scorpion species	t = 7.82, DF = 142 *p* < 0.0001 r^2^ = 0.301	t = 0.026, DF = 194 *p* = 0.979	t = 0.641, DF = 284 *p* = 0.522
AZ bark scorpion stinger	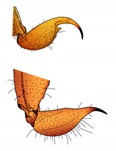	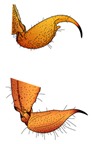	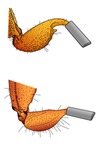
Stripe-tailed scorpion stinger			

## Supplementary Materials

The following are available online at https://zenodo.org/record/3762834#.XrZCPpm-uUm: Table S1: Body masses and energy content for a representative sample of AZ bark scorpions *(Centruroides sculpturatus)* and stripe-tailed scorpions *(Paravaejovis spinigerus)*, Tables S2–S4: Body masses of the scorpions of both species used in Experiments 1-3, Tables S5–S7: Preferences of individual grasshopper mice *(Onychomys torridus)* when choosing between briefly painful AZ bark scorpions and painless stripe-tailed scorpions in Experiments 1–3, Table S8: Handling times of the mice when feeding on both species of scorpions with and without “blocked” stingers, Table S9: Sex and mass for each of the 22 individual grasshopper mice used in Experiments 1–3, Video S1: Representative example of a single trial from Experiment 2, Video S2: Representative example of a single trial from Experiment 3.
